# Generation and Bioenergetic Profiles of Cybrids with East Asian mtDNA Haplogroups

**DOI:** 10.1155/2017/1062314

**Published:** 2017-09-28

**Authors:** Huaibin Zhou, Ke Nie, Ruyi Qiu, Jingting Xiong, Xiaoli Shao, Bingqian Wang, Lijun Shen, Jianxin Lyu, Hezhi Fang

**Affiliations:** ^1^Key Laboratory of Laboratory Medicine, Ministry of Education, Zhejiang Provincial Key Laboratory of Medical Genetics, College of Laboratory Medicine and Life Sciences, Wenzhou Medical University, Wenzhou, Zhejiang 325035, China; ^2^Zhejiang Provincial Hospital of TCM, Hangzhou, Zhejiang 310000, China; ^3^Hangzhou Medical College, Hangzhou, Zhejiang, China

## Abstract

Human mitochondrial DNA (mtDNA) variants and haplogroups may contribute to susceptibility to various diseases and pathological conditions, but the underlying mechanisms are not well understood. To address this issue, we established a cytoplasmic hybrid (cybrid) system to investigate the role of mtDNA haplogroups in human disease; specifically, we examined the effects of East Asian mtDNA genetic backgrounds on oxidative phosphorylation (OxPhos). We found that mtDNA single nucleotide polymorphisms such as m.489T>C, m.10398A>G, m.10400C>T, m.C16223T, and m.T16362C affected mitochondrial function at the level of mtDNA, mtRNA, or the OxPhos complex. Macrohaplogroup M exhibited higher respiratory activity than haplogroup N owing to its higher mtDNA content, mtRNA transcript levels, and complex III abundance. Additionally, haplogroup M had higher reactive oxygen species levels and NAD^+^/NADH ratios than haplogroup N, suggesting difference in mitonuclear interactions. Notably, subhaplogroups G2, B4, and F1 appeared to contribute significantly to the differences between haplogroups M and N. Thus, our cybrid-based system can provide insight into the mechanistic basis for the role of mtDNA haplogroups in human diseases and the effect of mtDNA variants on mitochondrial OxPhos function. In addition, studies of mitonuclear interaction using this system can reveal predisposition to certain diseases conferred by variations in mtDNA.

## 1. Introduction

Mitochondria are cytoplasmic organelles of eukaryotic cells with a double membrane and tortuous cristae structure that generate the bulk of ATP for cellular process including DNA decoding, kinase activation, and nutrient transport. ATP is produced in mitochondria via the oxidative phosphorylation (OxPhos) system, a functional unit located in the inner mitochondrial membrane. In mammals, the OxPhos machinery consists of five protein complexes—complex I (NADH:quinone oxidoreductase), complex II (succinate dehydrogenase), complex III (ubiquinol-cytochrome c reductase), complex IV (cytochrome c oxidase), and complex V (ATP synthase)—and two small molecules (coenzyme Q10 and cytochrome c). Components of all OxPhos complexes, with the exception of complex II, are mitochondrially encoded. Therefore, genetic variations in the mitochondrial genome can impact human health and disease.

Human mtDNA is double-stranded and circular with a length of 16,569 bp; it contains 37 genes, encoding 13 proteins of the OxPhos machinery and 22 tRNAs and two rRNAs for mitochondrial translation. mtDNA mutations cause changes in OxPhos system function, which have been implicated in various neuromuscular disorders such as Leigh syndrome and myoclonic epilepsy with ragged red fibers (MERRF) [1]. On the other hand, hundreds of mtDNA single nucleotide polymorphisms (SNPs) that can lead to nondeleterious alterations in OxPhos function have survived selective forces exerted by environmental factors and random drift during evolution [1].

A haplogroup is defined by variations in human mitochondrial DNA (i.e., SNPs) that are fixed and share a common ancestry. mtDNA haplogroups and their characteristic SNPs—for example, m.10398A/G [2], m.5178C/A [3], and m.152T/C [4]—can be advantageous or detrimental and have been implicated in a number of diseases and pathological conditions, including cancer [5], aging [6], diabetes [7], osteoarthritis [8], schizophrenia [9], and Leber's hereditary optic neuropathy [10]. A phylogenetic tree of L3 subhaplogroups that migrated from Africa to East Asia [11, 12] revealed that many of the subclades were associated with metabolic and degenerative diseases [13, 14]. The link between mtDNA haplogroup/SNPs and disease has been attributed to possible changes in mitochondrial reactive oxygen species (ROS) levels, respiration capacity, and ATP production [15, 16]. Functional analysis of mtDNA SNPs identified a common mtDNA variation, m.9821insA/AA, that affected mitochondrial ROS generation and OxPhos in mouse cells [17], while another showed that mtDNA haplogroups influence phenotypic variability in mice [18]. We and others have used transmitochondrial cytoplasmic hybrids (cybrids) to investigate the effects of mtDNA haplogroups/SNPs on disease conditions in human cells. Haplogroups related or unrelated to disease were fused with human 143B osteosarcoma cells lacking mtDNA rho zero (*ρ*0) cells [19], respectively. The effects of East Asian haplogroups/SNPs G versus B4 on osteoarthritis [15] and m.8584A/10398G versus m.8584G/10398A on Parkinson's disease [20] and of haplogroup B4 versus D4 on diabetes [14] and haplogroup B4 and E on biliary atresia [21] have been analyzed. However, mitochondrial function profile of the entire mtDNA phylogenetic tree was not reported.

To this end, in the present study, we used transmitochondrial technology to construct 21 cybrid cells representing different haplogroups to cover the entire Han Chinese mtDNA phylogenetic tree. The mtDNA phylogeny of the Han population, the largest ethnic group in China, is representative of that of all East Asian mtDNA phylogeny. We analyzed respiratory activity, mtDNA copy number, and retrograde signaling molecules in cybrid cells to define the spectrum of mitochondrial OxPhos in East Asian mtDNA haplogroups and to provide a foundation for mitochondria-based evolutionary medicine.

## 2. Materials and Methods

### 2.1. Generation of Cell Lines and Culture Conditions

143B *ρ*0 human osteosarcoma cells lacking mtDNA were cultured in high-glucose Dulbecco's modified Eagle's medium (DMEM, Thermo Fisher Scientific, Waltham, MA, USA) containing 10% fetal bovine serum (Thermo Fisher Scientific), 100 *μ*g/ml pyruvate, and 50 *μ*g/ml uridine. Cybrids were formed by fusing 143B *ρ*0 cells and platelets from healthy individuals with different haplotypes [15]. Cybrid clones were cultured in high-glucose DMEM containing 10% fetal bovine serum at 37°C in an atmosphere with 5% carbon dioxide.

### 2.2. mtDNA Sequencing, Genotyping, and Rebuilding the mtDNA Phylogenetic Tree

A total of 21 volunteers (mean ± SD: age 22 ± 0 years; 14 women and eight men) with different mtDNA haplotypes were recruited from the medical examination center of the First Affiliated Hospital of Wenzhou Medical University. Written informed consent was obtained from each participant. The study protocol was approved by the Ethics Committee of Wenzhou Medical University. Genomic DNA from the volunteers was extracted using sodium dodecyl sulfate (SDS) lysis buffer as described previously [8]. Sanger sequencing was performed for the entire mtDNA of each individual on an ABI 3730XL system (Thermo Fisher Scientific) with 24 primer pairs [15]. Detailed mtDNA haplotypes were annotated for each subject based on an established mtDNA tree [11, 12]. A phylogenetic mtDNA tree was reconstructed from the data derived from the 21 subjects according to an established East Asian mtDNA tree [11].

### 2.3. Quantification of mtDNA Copy Number

To determine mtDNA copy number, cells were incubated in SDS lysis buffer for more than 12 hours prior to genomic DNA extraction [22]. The ΔΔCT was used to determine the relative values of mtDNA copy number [23]. Briefly, Ct values generated from mtDNA were normalized to the housekeeping gene 18sDNA (2 [Ct mtDNA–Ct 18sDNA]). Relative mtDNA copy number for each cybrid was obtained by comparing to the average Ct value of mtDNA from 21 cybrids (2 [average Ct mtDNA−[Ct mtDNA–Ct 18S DNA]]). Real-time PCR was performed on an ABI Step-One Plus Real-Time PCR System (Thermo Fisher Scientific) using SYBR Green qPCR Mastermix (Takara Bio, Dalian, China) and the following primers were used: human mtDNA (human-tRNA leucine 1 + transcription terminator + 5S-like sequence), forward, 5′-CACCCAAGAACAGGGTTTGT-3′ and reverse, 5′-TGGCCATGGGTATGTTGTTAA-3′ and human nuclear DNA (18s ribosomal DNA), forward, 5′-TAGAGGGACAAGTGGCGTTC-3′ and reverse, 5′-CGCTGAGCCAGTCAGTGT-3′. The efficiency of the primers was 90%–110%.

### 2.4. Quantitative Reverse Transcriptase PCR Analysis

RNA was extracted from cybrids with Trizol reagent [24]. Total RNA (5 *μ*g) was reverse-transcribed into cDNA using an RNA reverse transcription kit (Takara Bio). Briefly, Ct values generated from mtRNA were normalized to that of the housekeeping gene 18S rRNA (2 [Ct mtRNA−Ct18srRNA]). The relative mtRNA level was obtained for each cybrid by comparing to the average Ct value of mtRNA from 21 cybrids (2 [average Ct mtRNA−[Ct mtRNA–Ct 18S RNA]]). Three sets of primers were used to determine the level of mtDNA transcripts started at the L, H1, and H2 promoters. The primer sets were as follows: (1) L strand: forward, 5′-GGTAGAGGCGACAAACCTACCG-3′and reverse, 5′-TTTAGGCCTACTATGGGTGT-3′ [25]; (2) H1 strand: forward, 5′-GGCCAACCTCCTACTCC-3′ and reverse, 5′-GATGGTAGATGTGGCGGGTT-3′ [26]; and (3) H2 strand: forward, 5′-AGCCACTTTCCACACAGACATC-3′ and reverse, 5′-GTTAGGCTGGTGTTAGGGTTCT-3′ [26]. Target mRNA levels were normalized against that of 18S rRNA. The 18S rRNA primers used were as follows: forward, 5′-GACGATCAGATACCGTCGTA-3′ and reverse, 5′-TGAGGTTTCCCGTGTTGAGT-3′. Data obtained from qRT-PCR were analyzed with the ΔΔCT method.

### 2.5. Mitochondrial Protein Preparation, Blue Native PAGE, and Immunoblotting

Mitochondrial proteins were isolated from cultured cells using 2% Triton-20 (Sigma-Aldrich, St. Louis, MO, USA) [27]. Proteins (30–60 *μ*g) mixed with 0.5% Blue G-250 (Sigma-Aldrich) and 5% glycerol were run on 3%–11% gradient blue native gels [27]. Antibodies against gene associated with retinoic-interferon-induced mortality 19, succinate dehydrogenase complex, subunit A, ubiquinol-cytochrome C reductase core protein 2, cyclooxygenase IV, and ATP synthase subunit 5*α* (all from MitoSciences, Eugene, OR, USA) were used to detect mitochondrial OxPhos complexes. Antibodies against voltage-dependent anion channel (Cell Signaling Technology, Danvers, MA, USA) and actin (Santa Cruz Biotechnology, Dallas, TX, USA) were used for loading controls. All primary antibodies were used at dilutions of 1 : 1000. Alkaline phosphatase- or horseradish peroxidase-conjugated anti-mouse IgG (both from Cell Signaling Technology) and anti-rabbit IgG (Thermo Fisher Scientific) secondary antibodies were used at 1 : 2000 dilution. Immunoreactivity was detected with the Super Signal West Pico chemiluminescence reagent (Thermo Fisher Scientific) or 5-bromo-4-chloro-3-indolyl phosphate/nitro blue tetrazolium substrates (Sigma-Aldrich). Signal of luminol-based enhanced chemiluminescence was detected using ChemiDoc imaging system (Bio-Rad, Hercules, CA, USA). Integrated optical density (IOD) quantification of immunoblots was performed using a Gel-Pro Analyzer 4.0 (Media Cybernetics, Warrendale, PA, USA).

### 2.6. Measurement of Endogenous Oxygen Consumption

Endogenous oxygen consumption of intact cells was determined using a Clark-type oxygen electrode (OROBOROS, Innsbruck, Austria) as described previously [28]. After recording basal respiration, oligomycin (0.1 mg/mL) (Thermo Scientific, Waltham, MA, USA) was added to detect uncoupling respiration. The standard protein content was measured using a BCA kit (Thermo Scientific, Waltham, MA, USA) to adjust experimental results.

### 2.7. Measurement of NAD^+^/NADH and Mitochondrial ROS

The NAD^+^/NADH ratio was detected in cultured cells with an NAD^+^/NADH ratio assay kit (Abcam, Cambridge, MA, USA) [15]. Briefly, cells were washed with phosphate-buffered saline (PBS) buffer and lysed with lysis buffer for 15 minutes at room temperature. Then, the NAD and NADH extraction reagents were added and the reaction was proceeded at room temperature for 15 minutes. PBS was used as blank control, and the fluorescence signal was measured after 30 minutes in conditions of excitation (540 nm) and emission (590 nm) with a Varioskan™ Flash Multimode Reader (Thermo Scientific, Waltham, MA, USA). Mitochondrial ROS were measured according to a previously published protocol [15]. Briefly, cells were washed with Hank's buffered salt solution (HBSS) and incubated with HBSS containing 5 *μ*M Mito SOX (Thermo Scientific, Waltham, MA, USA) at 37°C for 20 minutes. Cells were washed three times with HBSS, and fluorescence signal was detected using a Varioskan Flash Multimode Reader (Thermo Scientific, Waltham, MA, USA) under the conditions of excitation (488 nm) and emission (530 nm).

### 2.8. Fluorescence Microscopy of Mitochondrial Morphology

Cells were incubated with 500 nM MitoTracker Red (Thermo Scientific, Waltham, MA, USA) for 30 minutes and fixed for 15 minutes with 4% paraformaldehyde at room temperature. The cells were then permeabilized with 0.2% Triton X-100 (Sigma, St. Louis, MO, USA), stained with DAPI (Thermo Scientific, Waltham, MA, USA), and observed using an Olympus imaging system (Olympus FV1000, Melville, NY, USA). The length and complexity of the mitochondria were determined by measuring the form factor (FF) and aspect ratio (AR), respectively [29].

### 2.9. MMP Measurements

MMP was determined using the cationic fluorescent redistribution dye TMRM (Thermo Scientific, Waltham, MA, USA) as described previously [30]. Briefly, in the nonquenching mode, cells were incubated with 30 nM TMRM in a 37°C CO^2^ incubator for 30 min. Cells were washed three times with HBSS, and fluorescence signal was detected using a Varioskan Flash Multimode Reader (Thermo Scientific) under the conditions of excitation (350 nm) and emission (461 nm).

### 2.10. Statistical Analysis

Results are expressed as mean ± SEM and were analyzed with the Mann–Whitney *U* test using SPSS v.17.0 software (IBM, Armonk, NY, USA). Statistical analysis was performed only when specific SNPs were identified in three or more cybrids. For relative comparisons, results obtained from one cybrid were compared to the average value of all 21 cybrids. *P* < 0.05 was considered statistically significant.

## 3. Results

### 3.1. Generation of Cybrid Cell Lines Covering the Major East Asian mtDNA Haplogroups

**Figure 1:**
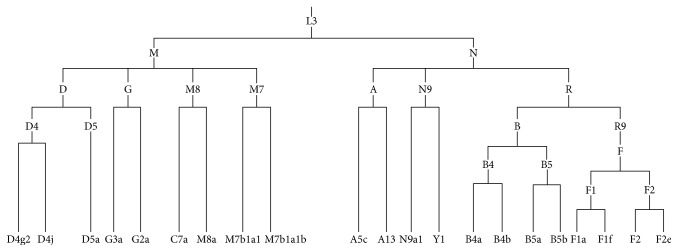
mtDNA tree of the 21 cybrids in China.

**Figure 2:**
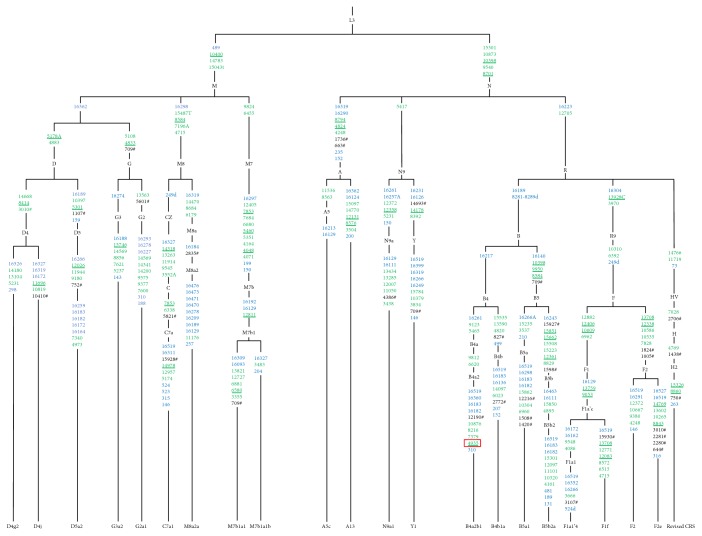
Relocation of mtDNA variants of 21 cybrids in the mtDNA tree. Blue and green colors represent control and coding regions, respectively. Nonsynonymous variants are shown in green and are underlined. The hatch marks indicate RNA variations, and the red box denotes novel mutations not reported in published databases. rCRS: revised Cambridge Reference Sequence.

To investigate the effects of East Asian mtDNA haplogroups on OxPhos, we generated cybrid cell lines containing different mtDNA haplogroups in the same osteosarcoma 143B nuclear background from Han Chinese, a population that comprises most East Asian haplogroup. Based on the mtDNA haplogroup classification tree [31, 32], 21 cybrid cell lines with D, G, M8, M7, A, N9, B, and R9 basal mtDNA haplogroups were generated to cover the common mtDNA macrohaplogroups of China, excluding two small clades [33], M9 and M10, enriched in Tibet (Figure 1). Four of the cybrid cell lines with haplogroups B4 and G were previously generated in our laboratory [15]. Entire mtDNA from cybrid cell lines was subject to Sanger sequencing and aligned to the mtDNA revised Cambridge Reference Sequence (rCRS, GenBank accession number NC_012920). An mtDNA phylogenetic tree was then reconstructed based on these sequencing results and the existing phylogenetic tree [12, 31, 32]. We detected 79, 160, and 31 variants in the D-loop, coding region, and tRNA/rRNAs, respectively, in the 21 cybrid cell lines (Figure 2 and Table S1 available online at https://doi.org/10.1155/2017/1062314). Most variants detected in this study can be retrieved as SNPs in published databases (e.g., mitomap, mtdb, and mtSNP) except m.4935G>A (Thr-Ala, ND2), a novel mutation of the B4a2b1 haplogroup identified in this study. However, this variant does not affect OxPhos function [15].

### 3.2. mtDNA Copy Number Variation in Different mtDNA Haplogroups

To analyze the effects of mtDNA haplogroups and their expression on the regulation of OxPhos complexes, we determined mtDNA copy number and transcriptional profiles by quantitative real-time PCR in the 21 cybrid cell lines. Nonsynonymous mutations were identified in 146 of the 270 identified SNPs. mtDNA copy number was lower in cybrids containing 4 SNPs (m.249del, m.13708A, m.13928C, and m.16304C) and higher in those containing the SNPs m.489C, m.8701G, m.10398G, and m.10400T as compared to than in cybrids containing rCRS bases (Table S2). It is worth noting that six of these eight SNPs (m.489C, m.8701G, m.13708A, m.13928C, m.16304C, and m.10400T) are unique diagnostic SNPs of a specific mtDNA haplogroup in East Asia, whereas 10398G and m.249del are representative SNPs of two more haplogroups, suggesting that a different mtDNA haplogroup contributed to the difference in mtDNA copy number. Additionally, m.16223T and m.16266A showed slightly higher and lower mtDNA copy number, respectively, as compared to cybrids containing rCRS bases. To be noted, most mtDNA copy number variations were minor (<30%).

**Figure 3:**
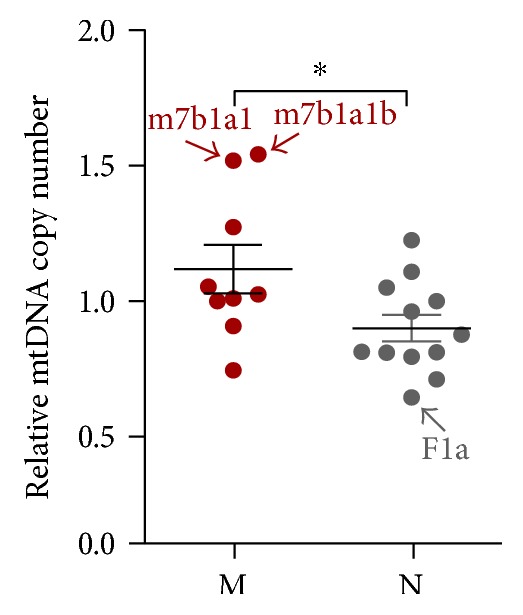
mtDNA copy number is higher in haplogroup M than in haplogroup N. Scatter plots represent mtDNA level relative to the average value for 21 cybrids. Data represents mean ± SEM of at least two independent experiments with 3 replicates each per cybrid. ^∗^*P* ≤ 0.05.

We analyzed macrohaplogroups M and N using marker SNPs unique to East Asian haplogroups. mtDNA copy numbers were 25% higher in cybrid cell lines with haplogroup M than in those with haplogroup N (Figure 3). The higher mtDNA copy number in haplogroup M was largely due to subhaplogroups M7b1a1 and M7b1a1b (both contributing a 1.51-fold difference) (Figure 3). Conversely, cybrids with subhaplogroup F1a containing m.16162G exhibited lowest mtDNA copy number and contributed to the greatest degree to the lower mtDNA copy number of haplogroup N (Figure 3). In addition, diagnostic SNPs of haplogroup M (m.489C, m.10398G, and m.10400T) showed a significantly higher mtDNA copy number than cybrids containing rCRS bases (Table S2).

### 3.3. Profiles of mtRNA and OxPhos Complex Levels in Cells with Different mtDNA Haplogroups

Next, we evaluated the expression of mtRNA in cybrid cells with different haplogroups. By measuring the mRNA levels of 16S sRNA, ND1, and 7S RNA from the primary transcripts of H1, H2, and L strands, respectively. The L strand encodes ND6 and eight tRNAs; its relative mRNA levels varied from 0.28 to 4.36, which were higher for m.16362C than for m.16362T (Table S3). H1 encodes 12S rRNA, 16S rRNA, and two tRNAs; relative H1 strand transcript levels varied from 0.47 to 2.17, which were lower for m.3010A than for m.3010G, while higher H1 transcript levels were observed for m.709A than for m.709G (Table S3). Relative transcript levels of the H2 strand, which encodes 12 tRNAs and 12 OxPhos subunits, varied from 0.56 to 1.74. Although H2 transcript levels were higher in cybrids containing the five SNPs m.489C, m.8701G, m.10398G, m.10400T, and m.16223T and lower in those containing m.16519C versus their rCRS bases (Table S3), H2 strand transcripts showed fewer variations than L and H1 strands. These results suggest that mtDNA transcription did not change significantly during mtDNA evolution. Additionally, most mtRNA transcription-related SNPs were diagnostic SNPs of mtDNA haplogroups.

**Figure 4:**
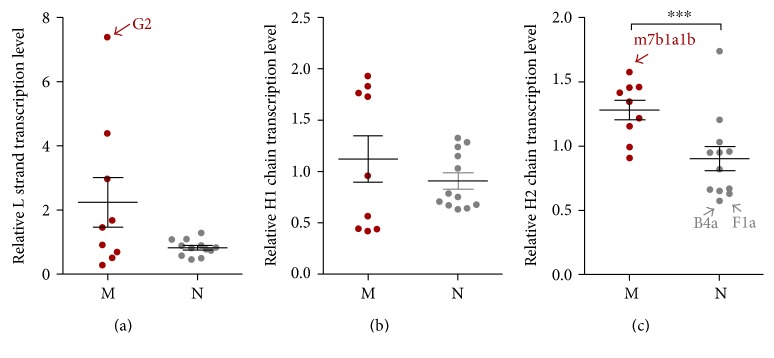
mtDNA transcript levels are higher in haplogroup M than in haplogroup N. Relative mtRNA expression was quantified by quantitative real-time PCR. The mRNA levels of 16S rRNA, ND1, and 7S RNA from the primary transcript represent H1, H2, and L strands of mtDNA, respectively. (a) Increased relative transcript level of mitochondrial L strand in haplogroup M. (b) Relative level of mitochondrial H1 strand transcript. (c) Higher relative transcript level of mitochondrial H2 strand in haplogroup M than in haplogroup N. Data represent mean ± SEM of two independent experiments with 3 replicates each per cybrid. ^∗∗∗^*P* < 0.001.

For haplogroups, we found that both L strand and H1 strand transcripts did not differ significantly between macrohaplogroups M and N (Figures 4(a) and 4(b)). However, L strand in haplogroup G2a is extremely higher than other haplogoups (Figure 4(a)). Transcriptional levels of the H2 strand in cybrid cell lines with haplogroup M increased significantly compared with those of haplogroup N (Figure 4(c)). Furthermore, M7b1a1b had the highest H2 transcripts in macrohaplogroup M, whereas the H2 transcripts in B4a and F1a were lowest in macrohaplogroup N (Figure 4(c)). However, the average increase of H2 transcripts in haplogroup M could be less than 20% compared with that of haplogroup N. Taken together, these results show that mtDNA genetic background does affect mtRNA transcription, but that the effect is limited.

**Figure 5:**
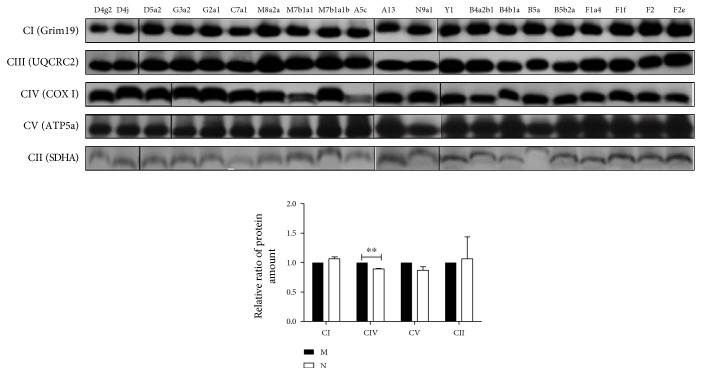
Respiratory chain complex III protein expression is higher in haplogroup M than in haplogroup N. Whole cell extracts of mitochondrial respiratory complex from 21 cybrid cell lines were solubilized in Triton-100 at a final concentration of 2% and subjected to blue native PAGE/immunoblot analysis. Complex I, complex II, complex III, complex IV, and complex V proteins were detected with antibodies against gene associated with retinoic-interferon-induced mortality (Grim19), succinate dehydrogenase complex, subunit (SDHA), ubiquinol-cytochrome C reductase core protein (UQCRC2), cyclooxygenase (COXIV), and ATP synthase subunit (ATP5*α*), respectively. Data represent mean ± SEM (*n* = 3). ^∗∗^*P* < 0.01.

Next, we examined individual OxPhos complexes I, III, IV, and V components using blue native PAGE in Triton X-100 solubilized whole cells and Western blot analysis. Both mtDNA copy number and mtRNA transcription were significantly affected in cybrid cells with haplogroups M and N. Moreover, our analysis showed that the level of complex III was significantly higher in haplogroup M than in haplogroup N, while complexes I, IV, and V were similar between these two haplogroups (Figure 5).

### 3.4. Mitochondrial Function in Cells Harboring Different mtDNA Haplogroups

To determine whether mitochondrial function in cybrid cells was affected by difference in mtDNA, mtRNA, and OxPhos complex profiles, we measured endogenous oxygen consumption in cells harboring different mtDNA haplogroups. The respiration assays revealed that seven SNPs significantly affected basal mitochondrial respiration. Of these, six and one SNPs were associated with lower and higher respiration activity, respectively, as compared with cybrids containing rCRS bases (Table S4). It is worth noting that three SNPs related to high basal respiration were diagnostic SNPs of haplogroup M (m.489C, m.8701G, and m.10400T). Basal respiration rates in cybrids harboring these seven SNPs ranged from 8.36 to 20.62 nmol/min/mg protein. Furthermore, oxygen consumption in the presence of oligomycin, an ATPase proton channel blocker, showed that four (m.489C, m.8701G, m.10400T, and m.16223T) and one (m.16519C) SNPs associated with higher and lower respiration, respectively, as compared with cybrids containing rCRS bases (Table S4).

**Figure 6:**
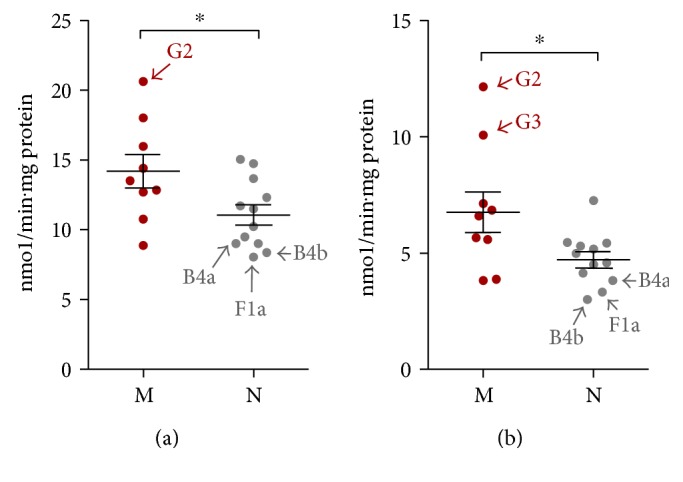
Oxygen consumption rate (OCR) is higher in haplogroup M than in haplogroup N. OCR values were measured in 21 cybrid cell lines after sequential treatments with oligomycin (0.1 mg/ml) and the OxPhos uncoupling agent trifluoromethoxy carbonyl cyanide phenylhydrazone (FCCP; 0.1 *μ*M). (a, b) Comparison of basal (a) and uncoupled (b) mitochondrial respiratory activity. Values represent mean ± SEM of at least two independent replicates. ^∗^*P* ≤ 0.05.

Basal mitochondrial respiration in cybrid cell lines with haplogroup M (m.489C, m.8701G, m.10400T, and most of m.10398G) was higher than that in cybrid cells with haplogroup N (Figure 6(a)). Furthermore, haplogroup G2 had the highest whereas B4b, B4a, and F1a had the lowest basal mitochondrial respiration rates in macrohaplogroups M and N, respectively. In addition, the degree of OxPhos uncoupling was higher in haplogroup M than in haplogroup N (Figure 6(b)). In haplogroup M, this was most apparent in subhaplogroup G2/3, which explains why “uncoupling mtDNA haplogroup” G was enriched in Siberia (Figure 6(b)). Accordingly, uncoupling respiration in B4b, B4a, and F1a was lowest in haplogroup N, contributing significantly to the respiration in macrohaplogroup N.

**Figure 7:**
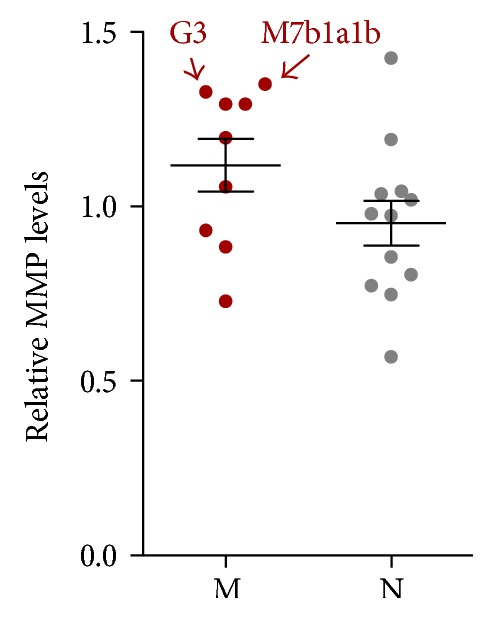
MMP is higher in haplogroup M than in haplogroup N. MMP levels were measured in cells treated with 30 nM tetramethylrhodamine (TMRM) for 30 min. Relative MMP levels were calculated by comparing reads obtained from the plate reader to the average value for all 21 cybrids. Data represent mean ± SEM of two independent experiments with 3 replicates each per cybrid.

To determine whether OxPhos function was altered, we measured mitochondrial inner membrane potential (MMP) in cybrid cell lines. The MMP of haplogroup M was slightly higher than that of haplogroup N (Figure 7), but this difference was not statistically significant (*P* = 0.11).

### 3.5. Mitochondrial Fragmentation in Cybrid Cells with Different mtDNA Genetic Background

**Figure 8:**
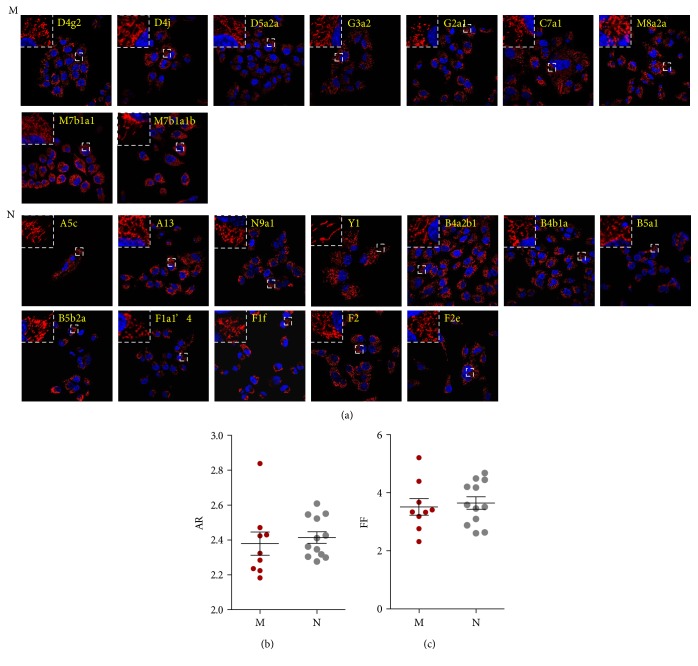
Morphometric analysis of mitochondria in cybrid cells. (a) Confocal micrographs of 21 cybrid cells in which the mitochondria were stained with MitoTracker Red (*n* = 3. Images are shown at 600x magnification. The upper and lower two rows show cybrid cells with macrohaplogroups M and N, respectively. mtDNA haplogroups are shown in yellow color. (b, c) Mitochondrial fragmentation was determined by AR (b) and FF (c), with higher values representing increased mitochondrial length/width and branching, respectively.

To investigate differences in mitochondrial morphology, mitochondria were stained with MitoTracker and visualized by confocal microscopy. Mitochondria fragmentation was determined by measuring the AR and FF of each mitochondrion, with high AR and FF reflecting increased mitochondrial length/width and branching, respectively. We found that AR was unaffected by mtDNA SNPs (Figure 8(a) and Table S5) and that most variations in these SNPs were limited (~10%). However, we identified two SNPs that slightly affected mitochondrial fragmentation (*P* < 0.01) (Figure 8(a) and Table S6). Thus, the percentage of mitochondrial fragmentation did not differ between mtDNA macrohaplogroups M and N (Figures 8(a), 8(b), and 8(c)).

### 3.6. Measurement of Intracellular Mitochondrial Signals in Cybrid Cell Lines

**Figure 9:**
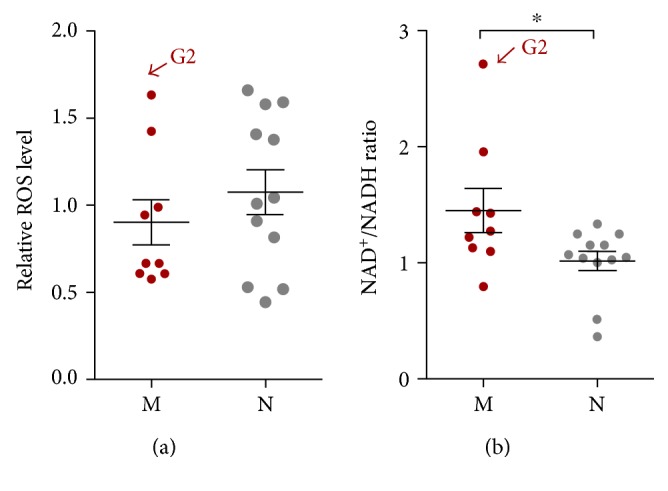
Measurement of intracellular mitochondrial signals in cybrid cell lines. (a) Comparison of intracellular ROS levels in cybrid cells. ROS levels were determined by staining cells with dichlorofluorescin diacetate. (b) NAD^+^/NADH ratio was higher in cybrid cells with haplogroup M than in those with haplogroup N. NAD^+^ and NADH levels in cell extracts were quantified based on fluorescence. Relative ROS levels were calculated by comparing reads obtained from the plate reader to the average value for all 21 cybrids. Data represent mean ± SEM of three independent experiments with 3 replicates each per cybrid. ^∗^*P* < 0.05.

We and others previously reported that mitochondrial haplogroups play an important role in mitonuclear interaction [15, 18, 34], in which, signals such as ROS, NAD^+^/NADH, and adenosine monophosphate activate or suppress retrograde signaling pathways in the nucleus. We found here that ROS levels did not differ significantly between macrohaplogroups M and N in the 21 cybrid cell lines (Figure 9(a)). Overall mitochondrial function and NAD^+^/NADH ratio were higher in cybrid cells with haplogroup M than haplogroup N (Figure 9(b)). However, there were no differences in ROS levels and NAD^+^/NADH ratio in haplogroups G2, B4, and F1a corresponding to the changes in mitochondrial function shown in Figure 6.

## 4. Discussion

Molecular epidemiology studies of mtDNA SNPs/haplogroups have revealed the contribution of mtDNA genetic background to human degenerative disease. The underlying etiological causes and mechanisms by which mtDNA haplogroups influence degenerative diseases are frequently investigated using cybrid technology. This approach allows us to establish multiple stable cell lines containing different mtDNA haplogroups in the same nuclear background [15, 35, 36]. Generally, cybrid-based studies examine all or some of mtDNA copy number, mtRNA expression level, OxPhos enzyme abundance and activity, and mitochondrial bioenergetics including respiration capacity, ATP generation, and MMP [15, 37–40]. In this study, we successfully generated 21 cybrid cell lines covering most East Asian haplogroup branches of the mtDNA phylogenetic tree. Previous cybrid analyses of mtDNA haplogroups in Taiwanese populations have been undertaken to examine the effect of mtDNA haplogroups B4/B5 in Parkinson's disease [41, 42] and B4/D4/N9 in diabetes [14, 38]. However, analyses of East Asian haplogroups and how they influence mitochondrial function have been limited. Thus, our system provides the first description of the spectrum of mitochondrial alterations that occur with different mtDNA haplogroups in East Asia. Secondly, our results provide a guideline to pinpoint specific disease-related risk/protective mtDNA haplogroups in molecular epidemiology studies. Lastly, we have established a comprehensive platform to further investigate the effect of mtDNA haplogroups on retrograde signaling at transcriptomic, proteomic, and metabolomic levels [18].

Here, we first described the differences in OxPhos function observed in different mtDNA genetic backgrounds from East Asia at the DNA, RNA, and protein level. Copy number analysis showed that 8 SNPs, m.249del, m.13708A, m.13928C, m.489C, m.8701G, m.10398G, m.10400T and m.16304C, might influence mtDNA replication, while others such as m.16362C may bear minor influence. Of which, m.489C, m.10398G, and m.10400T are genetically associated characteristic SNPs of macrohaplogroup M. These results are consistent with the observations that particular mtDNA haplogroups, with characteristic control region mutations, contribute to mtDNA replication [43, 44]. m.13708A and m.13928C, which exhibited lower mtDNA copy numbers than other SNPs, were found to be associated with increased risk of various diseases and pathological conditions [45, 46], whereas m.489C, m.10398G, and m.10400T with higher mtDNA copy numbers than rCRS bases had protective effects against disease [47, 48]. In addition, the disease-related SNPs m.489C, m.8701G, m.10398G, and m.10400T, as well as m.16362C/m.709A/m.16223T and m.3010A/m.16519C were associated with higher and lower mtRNA expression levels, respectively, than their rCRS bases. This suggests that these SNPs play a role in degenerative disease and should be included in molecular epidemiology studies.

Functional analysis of respiratory activity in cybrid cells confirmed that SNPs such as m.709A/m.16223C and m.13708A/m.16519C were associated with higher and lower endogenous mitochondrial respiratory activity, respectively, than their rCRS bases. Other SNPs, such as m.16362T>C, m.150C>T, and a 9 bp deletion, were shown to affect uncoupled mitochondrial respiration in the present study. However, no epidemiological studies have established a link between these SNPs and human degenerative disease. One limitation of our study is that nonhaplogroup defining SNPs were not examined, although the effects of SNPs from other sites cannot be ruled out by logistic regression with limited sample size. Additionally, an induced point mutation target to the candidate SNP may help to elucidate the function implication of specific SNP [49].

We also examined the effect of East Asian mtDNA haplogroups on mitochondrial function. Here, we focused on two macrohaplogroups M and N, while haplogroups M and N are the only two subhaplogroups of L3 immigrated from Africa into Eurasia during evolution. In Han Chinese, haplogroups M and N settled down and gave birth to their sublineages with the frequencies of 40–50% and 50–60%, respectively [11]. It is important to note that m.10398G represents haplogroup M and haplogroup B5, a subhaplogroup of N bearing a back mutation at m.10398. Cells with haplogroup M and m.10398G showed increased mtDNA copy number, L and H2 strand transcription, and respiratory chain complex III expression. Furthermore, cells bearing haplogroup M and m.10398G exhibited higher mitochondrial respiration (both coupled and uncoupled) than cells with haplogroup N and m.10398A, respectively. However, mitochondrial morphology did not differ between haplogroups M and N, nor between m.10398G and m.10398G A. Although it has been suggested in previous reports, ours is the first study to analyze the specific mitochondrial biogenetic profiles between haplogroups M and N [50]. mtDNA haplogroups M/N and related SNPs such as m.489T/C, m.8701A/G, m.10398A/G, and m.10400C/T, have been implicated in various pathological conditions. For example, mtDNA haplogroup M, m.10398G, and m.10400T were found to protect against aging [51], Alzheimer's disease [52], and Parkinson's disease [20], whereas mtDNA haplogroup N, m.10398A, and m.10400C are considered as risk factors. Whether haplogroups M and N are protective or risk-associated depends on the condition [2]. In fact, both mitonuclear interactions (retrograde signaling) and mitochondrial OxPhos function contribute to the development of disease [15, 18, 34], while variations in mitonuclear interactions have been reported in the two mtDNA haplogroups [15, 53]. Our data showed that the ratio of NAD^+^/NADH, a redox and energy stress marker, differed significantly between haplogroups M and N (Figure 9(b)), supporting the notion that haplogroups M and N exhibit distinct mitonuclear interaction patterns. However, mitochondrial ROS level did not differ between the two haplogroups (Figure 9(a)). Notably, subhaplogroup G of haplogroup M and subhaplogroups F1a and B4 of haplogroup N frequently reflected the functional differences between M and N in our analyses, suggesting that they play critical roles in degenerative disease. In addition, cell metabolism may be similarly affected by the same mtDNA with nuclear backgrounds from different races [18, 54]. Although our results indicate that haplogroups M and N differentially affect disease development, the use of Caucasian nuclear background-based cybrid system in the current work may limit the significance of this study, and further studies using *ρ*0 cells with Asian background are required to clarify this relationship.

## 5. Conclusions

In summary, we generated a cybrid-based system to investigate the mechanisms underlying the functional significance of mtDNA haplogroups that can provide molecular level insight into the role of mtDNA variants in human disease at the molecular level.

## Supplementary Material

Table S1. Analysis of whole mitochondrial genome. Table S2. Analysis of mtDNA copy number in 146 SNPs. Table S3. Analysis of mitochondria DNA transcription in 146 SNPs. Table S4. Analysis of endogenous oxygen consumption in 146 SNPs. Table S5. Analysis of aspect ratio of confocal images in 146 SNPs. Table S6. Analysis of form factor of confocal images in 146 SNPs.
